# Public perceptions of AI science and scientists relatively more negative but less politicized than general and climate science

**DOI:** 10.1093/pnasnexus/pgaf163

**Published:** 2025-06-17

**Authors:** Dror Walter, Yotam Ophir, Patrick E Jamieson, Kathleen Hall Jamieson

**Affiliations:** Department of Communication, Georgia State University, Atlanta, GA 30303, USA; Department of Communication, University at Buffalo, Buffalo, NY 14260, USA; Annenberg Public Policy Center, University of Pennsylvania, Philadelphia, PA 19104, USA; Annenberg Public Policy Center, University of Pennsylvania, Philadelphia, PA 19104, USA

**Keywords:** artificial intelligence, self-presentation of science, funding science, communicating science, scientific norms

## Abstract

Using a weighted 2023–2025 national probability panel of US adults, we compared the perceived Credibility, Prudence, Unbiasedness, Self-Correction, and Benefit (i.e. Factors Assessing Science's Self-Presentation [FASS]) of AI scientists with those of scientists in general and climate scientists in particular. Our analysis reveals that respondents’ composite perceptions of AI scientists are the most negative of the three, a difference driven by a facet of the Prudence factor, specifically the perception that AI science is causing unintended consequences; political ideology and patterns of media exposure are substantially more predictive of perceptions of climate science and science in general than of AI; and FASS and respondent ideology predict more variance in support for federal funding of the other two than of AI.

## Introduction

Perceptions of new technologies can shape public acceptance, funding, and policy (1, 2), as can politicized perceptions of science and individual scientific domains (e.g. climate science (3, 4) and medical science (5)). Partisan media coverage has also been found to correlate with perceptions and contribute to polarization of science (1 ). Whether warranted or not, public concerns or politicization of an emergent scientific domain can slow acceptance of the knowledge (e.g. climate change science) (1 ) or applications (e.g. genetically modified food (6) or COVID-19 vaccination (7)) it generates and potentially affect confidence in science itself (8).

Scientists have featured both the promise and perils of the rapidly evolving scientific area of AI (9 ). Media coverage of AI and its applications, such as ChatGPT, has been increasingly negative, with a growing focus on their problematic consequences and byproducts (10). Yet, the National Science Foundation's Science and Engineering Indicators February 2024 report notes that “much about human perceptions of AI remains undocumented” (11).

To provide a template for assessing public perception of AI science over time and to ascertain whether perceptions of it have polarized, we employ the Factors Assessing Science's Self-Presentation (FASS) scale (2 ) which determines whether science is perceived as Beneficial and Self-Correcting and scientists are perceived to be Credible, Unbiased, and Prudent. Past work has associated these factors with support for funding basic research and science in general (2) and science-consistent climate policy. We compare US public perceptions of AI with science in general (a benchmark that had been studied extensively in the past) (2) and climate science, a domain selected because perceptions of it are politically polarized (1).

Since the cost of scientific research is rising and the private sector is reluctant to fund noncommercializable research (1), we also examine the relationship between perceptions and support for federal funding of AI research.

## FASS across domains

**Fig. 1.**
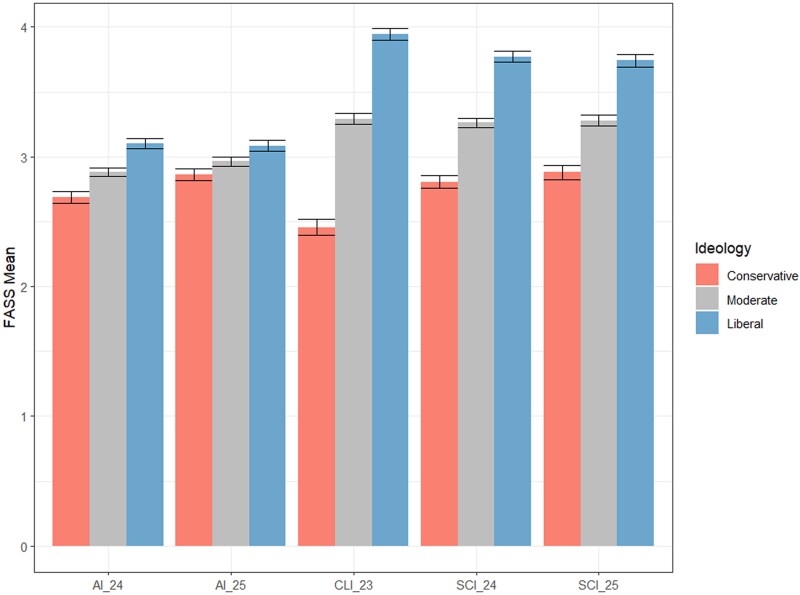
FASS means by scientific domain (AI, climate science, and science) and ideology (conservative to liberal), and FASS components.

As Fig. 1 indicates, FASS AI perceptions are comparatively negative. An ANOVA test revealed a significant difference between FASS perceptions across the three scientific domains (*F*(4, 3,662) = 37.54, *P* < 0.001). A Tukey post hoc test revealed significant differences between AI and climate science, and AI and science in general for both 2024 and 2025 (all *P* < 0.001), but not between science in general and climate science (*P* > 0.05 for both 2024 and 2025). Differences between perceptions of AI in 2024 and perceptions of AI in 2025 were not significant. Differences between science in 2024 and science in 2025 were not significant. In sum, AI was perceived consistently and significantly more negatively than climate science and science in general.

The analysis of individual scale items (Table 1) identified ‘unintended consequences’ as an AI perception driver. Participants gave AI scientists the lowest score on unintended consequences in both years (*M* = 2.263 for 2024 and *M* = 2.33 for 2025). The biggest differences between AI science and science in general in 2025 were for unintended consequences (Mean diff = 0.52), ‘share my values’ (Mean diff = 0.52), ‘benefit Me’ (Mean diff = 0.54), and ‘trustworthiness’ (Mean diff = 0.59). Comparing climate science (2023) with AI (2025), the biggest difference in means for the FASS items was for unintended consequences (Mean diff = 0.*66*), share my values (Mean diff = 0.64), trustworthiness (Mean diff = 0.*65*), and ‘unbiased in area’ (Mean diff = 0.52).

**Table 1.** Means and standard deviations of the 13 FASS items for AI science, science in general, and climate science.

	Climate 23	AI 24	AI 25	Science 24	Science 25
Competent	3.83 (0.04)	3.55 (0.04)	3.65 (0.03)	3.91 (0.03)	3.91 (0.03)
Trustworthy	3.62 (0.04)	2.99 (0.03)	2.97 (0.03)	3.60 (0.04)	3.56 (0.03)
Share values	3.31 (0.04)	2.65 (0.03)	2.67 (0.03)	3.22 (0.04)	3.19 (0.04)
Superior	3.05 (0.04)	2.69 (0.03)	2.70 (0.03)	2.72 (0.03)	2.75 (0.03)
Unintended consequences	2.99 (0.04)	2.26 (0.03)	2.33 (0.03)	2.93 (0.04)	2.85 (0.04)
Cut corners	3.12 (0.04)	2.70 (0.04)	2.80 (0.03)	2.89 (0.04)	2.91 (0.04)
Biased in area	3.31 (0.05)	2.76 (0.04)	2.79 (0.03)	3.30 (0.04)	3.24 (0.04)
Overcome bias	3.08 (0.04)	2.72 (0.04)	2.77 (0.03)	3.13 (0.04)	3.19 (0.04)
Benefit country	3.50 (0.04)	3.30 (0.03)	3.37 (0.03)	3.85 (0.04)	3.87 (0.03)
Benefit me	3.29 (0.05)	3.15 (0.04)	3.34 (0.03)	3.81 (0.04)	3.88 (0.04)
Mistakes caught	3.22 (0.04)	3.15 (0.03)	3.22 (0.03)	3.33 (0.04)	3.37 (0.04)
Responsibility taken	2.90 (0.05)	2.76(0.04)	2.85 (0.04)	2.87 (0.04)	2.96 (0.04)
Fraud prevented	3.19 (0.05)	3.07 (0.04)	3.11 (3.04)	3.16 (0.04)	3.17 (0.04)

## FASS and political ideology

Across ideologies (*F*(2, 3,652) = 322.12, *P* < 0.001), liberals had more positive perceptions across the three domains compared with moderates, and moderates had more positive ones compared with conservatives (all *P* < 0.001). The interaction between domain and ideology was significant (*F*(4, 3,652) = 19.79, *P* < 0.001), with ideological differences larger for climate science and science in general than AI. The difference between liberals and conservatives on FASS was 0.85 (2024, on a 5-point scale) and 0.85 (2025) for science in general, 1.32 (2023) for climate science, and 0.48 (2024) and 0.28 (2025) for AI.

An examination of the degree to which political ideology predicts FASS reveals that while the relationship is significant in all models (*P* < 0.001), variance explained is highest for climate science (adj. *R*^2^ = 0.31), followed by science in general (adj. *R*^2^ = 0.20 in 2024 and 0.17 in 2025), and AI science (adj. *R*^2^ = 0.07 in 2024 and 0.02 in 2025). Comparison of *r* values converted from raw (nonadjusted) *R*^2^ scores shows the difference between AI and science to be small (Cohen's *q* = 0.198 for 2024 and *q* = 0.290 for 2025) but significant (*P* < 0.001), and the difference between AI and climate to be moderate and significant (*q* = 0.345 for 2024 and *q* = 0.475 for 2025, *P* < 0.001).

## Media exposure and FASS

Controlling for ideology, patterns of media exposure were more predictive of perceptions of climate science (adj. *R*^2^ = 0.491), followed by science in general (*R*^2^ = 0.353 in 2024 and *R*^2^ = 0.377 in 2025), and least predictive for AI (*R*^2^ = 0.165 for 2024 and *R*^2^ = 0.067 for 2025). The difference between AI and science in general is small (*q* = 0.247 for 2024 and *q* = 0.434 for 2025) and significant (*P* < 0.001), and between AI and climate, small to moderate (*q* = 0.431 for 2024 and *q* = 0.584 for 2025; *P* < 0.001). A breakdown of relationships between media exposure to specific media outlets and perception of science over the three domains is reported in Table 2.

**Table 2.** Regression models predicting FASS per domain and year by media exposure and ideology.

	Climate 2023	AI		Science in general	
	2023	2024	2025	2024	2025
	*B* (SE)	*B* (SE)	*B* (SE)	*B* (SE)	*B* (SE)
Intercept	2.18 (0.09)***	2.3 (0.08)***	2.60 (0.09)***	2.46 (0.08)***	2.37 (0.09)***
Far-right	−0.19 (0.05)***	−0.04 (0.04)	−0.03 (0.05)	−0.09 (0.04)*	−0.10 (0.05)*
Centrist	0.20 (0.03)***	0.11 (0.02)***	0.09 (0.03)***	0.18 (0.02)***	0.22 (0.03)***
Social media	0.04 (0.03)	0.09 (0.02)***	0.07 (0.03)**	−0.04 (0.02)	0.05 (0.03)
Fox News	−0.13 (0.02)***	−0.04 (0.02)**	−0.02 (0.02)	−0.08 (0.02)***	−0.08 (0.02)***
Liberal	0.06 (0.02)**	0.01 (0.02)	0.02 (0.02)	−0.01 (0.02)	0.02 (0.02)
Science	0.07 (0.02)**	0.02 (0.02)	−0.03 (0.02)	0.06 (0.02)**	0.07 (0.02)**
Christian	0.02 (0.03)	0.06 (0.03)*	0.01 (0.04)	−0.00 (0.03)	0.06 (0.03)*
Alt-health	−0.07 (0.03)*	−0.08 (0.03)*	−0.03 (0.03)	−0.04 (0.03)	−0.14 (0.03)***
Ideology	0.25 (0.02)***	0.08 (0.02)***	0.05 (0.03)	0.15 (0.02)***	0.15 (0.03)***
Adj. *R*^2^	0.491	0.165	0.067	0.353	0.377

*P < 0.05, **P < 0.01, ***P < 0.001.

## Predicting support for federal funding using FASS

Broadly, the same FASS factors predicted support for funding across science in general, climate science, and AI science (Table 3), with one notable difference. Political ideology predicted support for funding of climate science and science in general, but not funding AI research. Importantly, FASS and ideology explained more variance in support for funding climate science (*R*^2^ = 0.609) than science in general (*R*^2^ = 0.417 for 2024 and *R*^2^ = 0.459 for 2025), and the least of the three for AI (*R*^2^ = 0.306 for 2024 and *R*^2^ = 0.199 for 2025). The difference between AI and science in general was small (*q* = 0.145 for 2024 and *q* = 0.369 for 2025) and significant (*P* < 0.001), and between AI and climate was small to medium (*q* = 0.423 for 2024 and *q* = 0.562 for 2025; *P* < 0.001).

**Table 3.** Regression models predicting funding from FASS factors and ideology.

	Climate 2023		AI				Science			
			2024		2025		2024		2025	
	*B* (SE)	*β*	*B* (SE)	*β*	*B* (SE)	*β*	*B* (SE)	*β*	*B* (SE)	*β*
Intercept	−0.38 (0.12)**		0.24 (0.16)		0.61 (0.22)**		0.35 (0.15)*		0.38 (0.14)**	
Unbiased	0.07 (0.05)	0.06	0.03 (0.05)	0.03	−0.04 (0.06)	−0.03	0.03 (0.05)	0.02	0.09 (0.05)	0.08
Credibility	0.42 (0.05)***	0.33	0.26 (0.06)***	0.19	0.24 (0.08)**	0.16	0.06 (0.07)	0.04	0.28 (0.06)***	0.23
Self-correct	0.16 (0.05)***	0.13	0.13 (0.05)**	0.10	−0.01 (0.05)	−0.01	0.13 (0.04)**	0.12	0.01 (0.05)	0.01
Benefit	0.18 (0.05)***	0.16	0.28 (0.05)***	0.24	0.39 (0.06)***	0.31	0.31 (0.05)***	0.27	0.18 (0.05)***	0.17
Prudent	0.14 (0.05)**	0.10	0.15 (0.05)**	0.09	0.20 (0.06)***	0.12	0.29 (0.05)***	0.21	0.20 (0.05)***	0.16
Ideology	0.21 (0.03)***	0.18	0.04 (0.03)	0.04	0.01 (0.03)	0.01	0.21 (0.03)***	0.20	0.23 (0.03)***	0.25
Adj. *R*^2^	0.609		0.306		0.199		0.417		0.459	

*P < 0.05, **P < 0.01, ***P < 0.001.

## Discussion

This study examines public perceptions of AI, while offering a blueprint for identifying and tracking respondent ideology and media exposure and perceptions of five factors (FASS) with the potential to affect perceptions of emerging scientific fields: perceived Credibility, Prudence, Unbiasedness, Self-Correction, and Benefit. While previous work has pointed towards a general negativity towards AI, our study harnesses the multifaceted FASS measurement to identify specific public concerns about AI science and scientists. Our findings suggest that on key FASS indicators, the public distinguishes AI from science in general and climate science. Participants expressed more negative views of AI science/scientists, with the strongest concern expressed about AI's potential to create unintended consequences. While this concern is consistent with the media's growing focus on the dangers of AI (10), future studies should examine whether the relationship between media exposure and perceptions is causal.

Since it is possible that negative perceptions result, in part, from AI's novelty, we examined changes between 2024 and 2025. This comparison showed public perceptions of AI science and scientists did not improve over time, even as more Americans adopted AI applications following the 2022 release of ChatGPT. In other words, our data suggest that negativity towards AI reflects more than just a passing moral panic in light of a novel technology (12). Nevertheless, a close examination of future trends in public perceptions is merited considering the rapid growth and development in AI technology.

Our study also examines how, if at all, ideology and media habits relate to perceptions of AI science. Some scientific domains, such as climate change, have long suffered from a partisan politicization in the United States. More recently, research identified a pandemic-associated decline in Republicans’ confidence in medical scientists and scientists in general (5), which raised the possibility that politicization of emerging COVID-related science affected the perceptions of science in general. Our finding that AI has (at least yet) not been politicized in the United States could guide future science communication around this technology. As with the general negativity towards AI science and scientists, ongoing monitoring of ideology and media exposure as possible predictors of perception of AI science remains a priority.

Our findings indicate that FASS, ideology, and media exposure are less predictive of support for federal funding of AI science compared with science in general and climate science. As such, we call for future research into other factors that may shape public perception of AI science and support of funding of research around this consequential technology.

Identifying negative perceptions and their antecedents can incentivize vigilance and guide science messaging. The public unease about AI's potential to create unintended consequences invites transparent, well-communicated ongoing assessment of the effectiveness of self or governmental regulation of it. At the same time, it is imperative to examine whether perceptions of AI result, in part, from novelty. New technologies are often met with skepticism and even moral panic (12) and so to reduce novelty bias, our study examines perceptions of AI over time as well.

## Materials and methods

Data were collected in four rounds via the Annenberg Science and Public Health survey using an online national probability panel survey drawn from a larger SSRS national probability panel of US adults aged 18 and older. All procedures were reviewed and approved by the University of Pennsylvania's Institutional Review Board and were deemed to meet the eligibility criteria for IRB review exemption authorized by 45 Code of Federal Regulations 46.104, category 2.

Media exposure and perceptions about science were measured in March 2023 (*n* = 1,638). Perceptions about climate science were measured in November 2023 (*n* = 1,538). Perceptions related to AI science and science in general were measured in February 2024 (*n* = 1,555) and February 2025 (n = 1,716). The study's identification of the political slant of media outlets (1) is consistent with the self-reported media preferences of partisans (13), academic studies of partisan citation patterns within media outlets (14), and evaluations by nonpartisan sources validated in previous studies (15).

Perceptions of science and scientists were measured using the 13 items FASS scale clustered into five factors: Credibility, Unbiased, Prudent, Beneficial, and Self-correcting (2 ) with all items measured from 1 (strongly disagree) to 5 (strongly agree). Each item was asked separately for science, climate science, and AI science. Reliability of the FASS model was estimated using Cronbach's alpha with values ranging from *α* = 0.600 to *α* = 0.953 with an average value of *α* = 0.818.

Details regarding the materials and methods are summarized in Appendix A. Question wording for items are summarized in Appendix B.

## Supplementary Material

pgaf163_Supplementary_Data

## Data Availability

Survey data and code are available at the Center for Open Science (OSF) in the following link: https://osf.io/gjchx/ (DOI: 10.17605/OSF.IO/GJCHX).
