# Supplementary material for: Public perceptions of AI science and scientists relatively more negative but less politicized than general and climate science
Source: PNAS Nexus. 2025 Jun 17;4(6):pgaf163. doi: 10.1093/pnasnexus/pgaf163 (PMC12199247; doi:10.1093/pnasnexus/pgaf163)
Supplement: pgaf163_Supplementary_Data [file pgaf163_supplementary_data.docx]

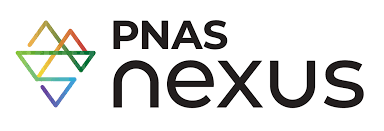


**Supporting Information for**

Public Perceptions of AI Science and Scientists Relatively More Negative but Less Politicized Than General and Climate Science

Dror Walter, Yotam Ophir, Patrick E. Jamieson and Kathleen Hall-Jamieson

Corresponding Author: Dror Walter

**Email:** dwalter2@gsu.edu

**This PDF file includes:**

Appendix 1: Materials and Methods

Appendix 2: Full wording of all survey questions used in the study

**Appendix 1: Materials and Methods**

Data were collected in three rounds via the Annenberg Science and Public Health survey (ASAPH) using an online national probability panel survey drawn from a larger SSRS national probability panel of U.S. adults aged 18 and older. All procedures were reviewed and approved by the University of Pennsylvania’s Institutional Review Board and were deemed to meet the eligibility criteria for IRB review exemption authorized by 45 Code of Federal Regulations (CFR) 46.104, category 2. SSRS Opinion Panel members are recruited randomly based on nationally representative ABS (Address Based Sample) design (including Hawaii and Alaska). ABS respondents are randomly sampled by Marketing Systems Group (MSG) through the U.S. Postal Service’s Computerized Delivery Sequence File (CDS), a regularly updated listing of all known addresses in the U.S. For the SSRS Opinion Panel, known business addresses are excluded from the sample frame.

For the first wave (2023, Climate) 1,538 respondents were surveyed (1,512 in English, 26 in Spanish; 1,502 using web interface, and 36 via telephone; Survey completion rate was 77.9%). For the second wave (2024, AI and General Science) 1,555 respondents were surveyed (1,526 in English, 29 in Spanish; 1,515 using web interface, and 40 via telephone; Survey completion rate was 79.2%). For the 2025 data (AI and General Science) A total of 1,716 panelists completed the survey. Full question wording for all items can be found in Appendix 2.

The data were weighted to balance the demographic profile of the sample to the target population parameters. The weighting for the first two waves was based on distributions of sex by age, sex by education, age by education, race/ethnicity, census region, civic engagement, population density, party ID, voter registration, religious affiliation, and internet use frequency. The main demographic benchmarks were obtained from the 2022 Annual Social and Economic Supplement (ASEC) of Current Population Survey (CPS). The civic engagement benchmark was derived from September 2021 CPS Volunteering and Civic Life Supplement data. Population density was derived from 2021 Census Planning Database. The registered voter benchmark is from Aristotle Voter Data 2022 and Annual Estimates of the Resident Population by Single Year of Age and Sex for the United States: April 1, 2020 to July 2, 2021 from the U.S. Census Bureau. The party ID, internet frequency, and religious affiliation benchmarks came from NPORS annual dataset released by Pew Research. Weights were trimmed at the 2nd and 98th percentiles to prevent individual interviews from having too much influence on survey-derived estimates.

The weighting for the third wave (2024) was based on distributions of: sex by age, sex by education, age by education, race/ethnicity, census region, civic engagement, population density, party ID, voter registration, religious affiliation, and internet-use frequency. The main demographic benchmarks were obtained from the 2023 Annual Social and Economic Supplement (ASEC) of Current Population Survey (CPS). The civic engagement benchmark was derived from September 2021 CPS Volunteering and Civic Life Supplement data. Population density was derived from the Claritas Pop-Facts Premier 2023. The registered voter benchmark is from Aristotle Voter Data 2023 and Annual Estimates of the Resident Population by Single Year of Age and Sex for the United States: April 1, 2020 to July 2, 2022 from the U.S. Census Bureau. The party ID, internet frequency, and religious affiliation benchmarks came from the 2023 NPORS annual dataset released by Pew Research Center. Weights were trimmed at the 4th and 96th percentiles to prevent individual interviews from having too much influence on survey-derived estimates.

For t2025 data were weighted to distributions of: sex by age, sex by education, age by education, detailed education, race/ethnicity, census region, home tenure, number of adults per household, civic engagement, population density, party ID, voter registration, religious affiliation, and internet use frequency. The main demographic benchmarks were obtained from the 2024 Annual Social and Economic Supplement (ASEC) of Current Population Survey (CPS). The civic-engagement benchmark was derived from the September 2023 CPS Volunteering and Civic Life Supplement data. Population density was derived from the Claritas Pop-Facts Premier 2023. The registered voter benchmark is from the 2023 Annual Social and Economic Supplement (ASEC) of Current Population Survey (CPS)’s 2022 Voting and Registration Supplement. The party ID, internet frequency, and religious affiliation benchmarks came from the 2024 NPORS annual dataset released by Pew Research Center.

Media exposure and perceptions about science in general were measured in March 2023 (n = 1,638). Perceptions about climate were measured in November 2023 (n = 1,538), and those related to AI and science in general in February 2024 (n = 1,555) and February 2025 (n = 1,716). The political slant of outlets^1^ was determined based on a combination of audience preferences^2^, academic studies^3^ and evaluations by the non-partisan sources, as used and validated in previous studies^4^.

Perceptions of science and scientists were measured using the 13-item FASS scale clustered into five factors (Credibility, Unbiased, Prudent, Beneficial, and Self-correcting)^9^ with all items measured from 1 (strongly disagree) to 5 (strongly agree). Each item was asked separately for science in general, climate science and AI science. Political ideology was measured from 1 (strongly conservative) to 5 (strongly liberal).

Lastly, support for federal funding of research was measured using two items. First, respondents were asked if federal funding of science should be increased, decreased or stay the same. For those that chose the first two replies, a follow-up question asked whether it should be increased/decreased significantly or somewhat. The two questions were combined to create a 5-point scale. Each item was asked separately for support for funding of science in general, and in the domains of climate and AI research.

| Table S1. Cronbach’s Alpha Values for the FASS Factors for all Contexts and Waves | | | | | |
| --- | --- | --- | --- | --- | --- |
|  | Climate 2023 | AI  2024 | AI  2025 | General Science 2024 | General Science 2025 |
| Unbiased | .863 | .741 | .728 | .817 | .792 |
| Credibility | .917 | .798 | .761 | .831 | .863 |
| Self-Correct | .877 | .794 | .811 | .821 | .842 |
| Benefit | .885 | .852 | .816 | .875 | .895 |
| Prudent | .782 | .600 | .606 | .734 | .703 |
| FASS | .953 | .884 | .873 | .921 | .926 |

**Appendix 2: Full wording of all survey questions used in the study**

The FASS components were measured using a 13-question scale. For all items possible answers ranged from (1) Strongly disagree to (5) Strongly agree.

The credibility component was measured using the following three items:

1. “[(Scientists)/(Scientists working on artificial intelligence)/(Climate Scientists)] are competent”
2. “[(Scientists)/(Scientists working on artificial intelligence)/(Climate Scientists)] are trustworthy”
3. “[(Scientists)/(Scientists working on artificial intelligence)/(Climate Scientists)] share my values”.

The prudence component was measured using three items:

1. “[(Scientists)/(Scientists working on artificial intelligence)/(Climate Scientists)] feel superior to others”,
2. “[(Scientists)/(Scientists working on artificial intelligence)/(Climate Scientists)] creates unintended consequences and replaces older problems with new ones”,
3. “[(Scientists)/(Scientists working on artificial intelligence)/(Climate Scientists)] do whatever it takes to get grants and publish, even if that means cutting corners”.

The unbiased component was measured using two items:

1. “[(Scientists)/(Scientists working on artificial intelligence)/(Climate Scientists)] provide the public with unbiased conclusions about their area of inquiry”,
2. “In general, when [(Scientists)/(Scientists working on artificial intelligence)/(Climate Scientists)] are doing their work, they are able to overcome human and political biases”.

Beneficial was measured using a two-item scale:

1. “Scientific findings produced by U.S. [(Scientists)/(Scientists working on artificial intelligence)/(Climate Scientists)] in the past decade have benefited the country as a whole”
2. and “Scientific findings produced by U.S. [(Scientists)/(Scientists working on artificial intelligence)/(Climate Scientists)] in the past decade have benefited people like me”.

Lastly, the self-correcting component was measured using three items:

1. “Just your best guess, when there are mistakes in [(research)/(artificial intelligence research)/(climate-change research)], how often are they caught by the peer-review process in which scientists evaluate each other’s work before it is published?”
2. “Just your best guess, when [(Scientists)/(Scientists working on artificial intelligence)/(Climate Scientists)] make a mistake, how often do they take responsibility for it?”
3. “Just your best guess, when fraud in [(research)/(artificial intelligence research)/(climate-change research)] is uncovered, how often do scientists take action to prevent other researchers from engaging in the same type of conduct?”

Ideology was measured on a 5-point scale ranging from (1) very Conservative to (5) very Liberal.

Support for funding was measured in two stages.

1. First, respondents were asked “Should federal funding for [(science)/(artificial intelligence Science)/(Climate Science)] be increased, decreased or stay about the same?”.
2. Then, based on their answer on the first item, they were asked “Should federal funding for [(science)/(artificial intelligence Science)/(Climate Science)] be [increased/decreased]: Significantly or Somewhat”.

Media consumption was measured using 15 items. For each items respondents were asked to rate the extent to which they receive (0) NO information, to (5) A LOT of information, from that media sources. For Far-right respondents estimated the amount of information the received from sources such as such as Newsmax, One America News (OAN), Gateway Pundit, Parler, Telegram, GETTR, Mark Levin, Breitbart News, and Truth Social. For centrist media we used legacy news sources such as CBS News, NBC News, ABC News, and CNN news, as well as legacy print sources such as the Associated Press, and the news pages of The Wall Street Journal, or the New York Times. For social media we used sources such as Facebook, Instagram, Tik-Tok and Twitter. Liberal media consumption was measured using items such as MSNBC and Huffington Post. Fox News consumption was measured using one item (Fox News). Science-oriented media was measured using science magazines or programs such as Nova, Popular Science, or Scientific American. Christian media as measured using Sources such as Christian Broadcasting Network (CBN), EWTN, or Daystar Television Network. Lastly, Alt-health sources was measured using Social media accounts dedicated to alternative health, such as Natural News or Earth Clinic.

The political slant of channels was determined based on a combination of polls showing audience preference, academic studies, and evaluations by the nonpartisan source, allsides.com, that was shown in recent studies to be consistent with other sources, including Ad Fontes, on the specific outlets we included (See Ophir et al., 2024).

We used Allsides.com to identify far-right sources, including Breitbart, Newsmax, and OAN. To them we added platforms that were created as hyper-conservative alternatives to mainstream social media and had become hubs for extremism, including Telegram, Gettr, Gateway Pundit, Truth Social, Parler, and Mark Levin’s shows.

While there are different ways to evaluate the exact slant of an outlet (e.g., whether MSNBC is strongly liberal or somewhat liberal), different methods agree on the general slant of the specific outlets we use here. Note that since the conservative bias of the Wall Street Journal is limited to its editorial sections, we asked participants about their exposure to the news pages specifically. Alternative health media was defined as “information sources that promote and amplify non-mainstream, non-standard treatments and health practices unsanctioned by such custodians of U.S. health information,” such as the CDC and the FDA.

**References**

1. Ophir, Y., Walter, D., Jamieson, P. E. & Jamieson, K. H. The Politicization of Climate Science: Media Consumption, Perceptions of Science and Scientists, and Support for Policy. J Health Commun 29, sup1, 18-27 (2024). doi:10.1080/10810730.2024.2357571

2. Grieco, E. Americans’ main sources for political news vary by party and age. *Pew Research Center* <https://www.pewresearch.org/short-reads/2020/04/01/americans-main-sources-for-political-news-vary-by-party-and-age/> (2020).

3. Groseclose, T. & Milyo, J. A Measure of Media Bias*.* Q J Econ, 120, 1191–1237 (2005).

4. Wang, R., Thimmanayakanapalya, S. S. & Ophir, Y. The growing partisan politicization of non-political online spaces: A mixed-method analysis of news app reviews on Google Play between 2009 and 2022. New Media Soc*,* 14614448241237765 (2024) doi:10.1177/14614448241237765.
